# One size fits none – a qualitative study investigating nine national quality registries’ conditions for use in quality improvement, research and interaction with patients

**DOI:** 10.1186/s12913-018-3621-9

**Published:** 2018-10-20

**Authors:** Vibeke Sparring, Emma Granström, Magna Andreen Sachs, Mats Brommels, Monica E. Nyström

**Affiliations:** 1 Department of Learning, Informatics, Management and Ethics, Medical Management Centre, Karolinska Institutet, SE-17177 Stockholm, Sweden; 2 Department of Public health and Clinical medicine, Epidemiology and Global health, Umeå University, SE-90187 Umeå, Sweden

**Keywords:** National quality registry, Clinical registry, Clinical database, Quality improvement, Registry-based research, Patient-centred care

## Abstract

**Background:**

Swedish National Quality Registries (NQRs) are observational clinical registries that have long been seen as an underused resource for research and quality improvement (QI) in health care. In recent years, NQRs have also been recognised as an area where patients can be involved, contributing with self-reported experiences and estimations of health effects. This study aimed to investigate what the registry management perceived as barriers and facilitators for the use of NQRs in QI, research, and interaction with patients, and main activities undertaken to enhance their use for these purposes. The aim was further to identify potential differences between various types of NQRs for their use in these areas.

**Methods:**

In this multiple case study, nine NQRs were purposively selected. Interviews (*n* = 18) were conducted and analysed iteratively using conventional and directed content analysis.

**Results:**

A recent national investment initiative enabled more intensive work with development areas previously identified by the NQR management teams. The recent focus on value-based health care and other contemporary national healthcare investments aiming at QI and public benchmarking were perceived as facilitating factors. Having to perform double registrations due to shortcomings in digital systems was perceived as a barrier, as was the lack of authority on behalf of the registry management to request participation in NQRs and QI activities based on registry outcomes. The registry management teams used three strategies to enhance the use of NQRs: ensuring registering of correct and complete data, ensuring updated and understandable information available for patients, clinicians, researchers and others stakeholders, and intensifying cooperation with them. Varied characteristics of the NQRs influenced their use, and the possibility to reach various end-users was connected to the focus area and context of the NQRs.

**Conclusions:**

The recent national investment initiative contributed to already ongoing work to strengthen the use of NQRs. To further increase the use, the demands of stakeholders and end-users must be in focus, but also an understanding of the NQRs’ various characteristics and challenges. The end-users may have in common a need for training in the methodology of registry based research and benchmarking, and how to be more patient-centred.

## Background

There has been an increasing international interest in developing, improving and evaluating healthcare systems not only in terms of service, e.g. availability, but also in terms of other outcomes relevant to creating value for the different stakeholders, above all for the patients [1–3]. One source of information are clinical registries, which by collecting data about medical interventions, procedures and outcomes, monitoring adherence to national guidelines, and benchmarking performance between health care providers at various levels can be instrumental in improving the quality of health care [4]. The Swedish national quality registries (NQRs) are observational clinical registries containing information about two important knowledge systems involved in improvement, i.e. “generalizable scientific evidence” and “performance measurement” [5]. NQRs have been stated to improve quality of care [6, 7] and to enable fact-based decisions by patients, clinicians and managers about better care [8]. Information in the NQRs can also be used for new and more efficient research. The development of NQRs have contributed to Sweden’s strong position in the areas of acute cardiac care, diabetes care and hip replacement surgery, and among the best survival rates after heart attack, stroke, breast and colorectal cancer [9]. Altogether, these possibilities provide strategies for building learning healthcare systems [4, 10]. NQRs have also been recognised as a way to follow-up on the effects on decisions at a policy level and the performance of the healthcare system [11]. National clinical-quality registries have been developed in Australia [12], and the Swedish NQRs may inspire other countries as well [11].

The Swedish NQRs have also long been seen as a “largely untapped resource” [4], especially when it comes to using the registries for research but also for quality improvement (QI) [11, 13, 14]. In recent years, patient involvement and self-reported experience have been recognised as a gap in quality registries. The use of clinical quality registries for QI in health care has been of interest also outside Sweden (e.g., [15–18]). However, the effective use of clinical registry data seems to be hampered by shortcomings in design of the digital systems [10, 19], the lack of engagement by local staff and managers [19], and low interest among researchers for using quality registries for research purposes [4, 20].

To fulfil the identified potential of the NQRs more knowledge on how their use can be enhanced is needed. An opportunity for such studies arose when a five-year national investment initiative was launched in 2012 in order to promote the use of the Swedish NQRs for clinical development and QI, patient interaction, and research. The Swedish Government and the authorities responsible for healthcare jointly invested more than SEK 1.5 billion on the development of the NQRs. Accordingly, the aim of this study was to investigate what the registry management perceived as barriers and facilitators for the use of NQRs in QI, research, and interaction with patients, and the main activities undertaken to enhance their use for these purposes. The aim was further to identify potential differences between various types of NQRs for their use in these three areas.

## Methods

A multiple case study design [21] based on analyses of interviews was used to investigate the NQR’s conditions and strategies. This approach is considered relevant for research into open systems where events, processes and context cannot be controlled, as in this study [21]. We employed an inductive approach when analysing the data, but as we were guided by previous research on quality registries as well as our own experience in managing clinical databases, we applied content analysis rather than a theory generating technique.

### Setting

In Sweden, the responsibility for financing and organising health services lies upon the 21 counties and regions. Services are tax-funded and provided mainly by public providers, but the share of private services with public funding has increased, especially in primary health care. In the Swedish setting a care commissioner is “a state authority, county council and municipality in the case of such health and medical care that an authority, county council or municipality is responsible for, and other legal entity or individual entrepreneur who carries out health and medical care” [22]. In this decentralised system, the national government uses laws and regulations, but also soft governance measures such as national guidelines and national initiatives to steer the healthcare sector [23].

### National quality registries in Sweden

In Sweden, quality registries collect information from registry users to calculate and report on quality of care indicators. In contrast to internal quality development systems, NRQs collect information from numerous care providers. The first national quality registry, initiated in 1975 by orthopedic surgeons, was the Swedish Knee Arthroplasty Register. It has since then been followed by an increasing number of registries, today more than 100, focusing on specific disorders or healthcare processes [8]. Three certification levels were introduced in 2012 to assess the maturity and quality of a registry based on a set of indicators [14]. The certification process is led by an executive committee appointed by the Swedish Association of Local Authorities and Regions (SALAR). Members of the committee represent different stakeholders in Swedish health care and are responsible for allocation of funding.

A quality registry is a structured and automated collection of information about patients, initiated in order to develop and secure care quality and to make comparisons on regional and national level [24] and can also be used for research. Each registry must have a county council as legally responsible, and are subject to the laws regulating government authorities [8]. Registries consist of a steering group, a registry holder and a registry management team (including e.g. coordinator, administrator).

Since the 1990s, registries have been able to apply for funding from national agencies such as the National Board of Health and Welfare and SALAR. In 2012, a national initiative was launched to enhance the use of NQRs for clinical development and QI, patient interaction, and research. During 5 years (2012–2016), the Swedish Government and the authorities responsible for healthcare jointly invested just over SEK 1.5 billion (in excess of the regular funding) to further develop the NQRs. Six regional Quality Registry Centres (QRCs) were also financed within this initiative to support NQRs to reach the goals of the initiative [25].

The original purpose of the NQRs was to agree on and spread best practice. Today, the purpose has expanded to encompass research and benchmarking, which has put an emphasis on the need to guarantee the quality of the NQRs. On a national, regional or hospital level, aggregated data is compiled to compare different care providers’ results or outcomes. To support the improvement of local clinical practices, data are or need to be real-time and fed back to practitioners [26]. Furthermore, fruitful interaction with different stakeholders requires that relevant data are collected and made available for those target groups, mainly clinicians, decision-makers on different levels, and patients.

### Selection of NQRs

The selection was done iteratively in several steps in order to obtain a purposive sample. The following steps and criteria were applied. Firstly, having a certification level of 1 or 2 served as an inclusion criteria. At the time of the selection (Feb, 2015), 41 registries were certified at level 1 or 2. Secondly, NQRs were selected to show wide variation based on type of registry [8, 20] ensuring that at least one from the following categories were included: intervention registry; diagnosis registry; and registry with a focus on prevention, palliative care, or psychiatry. Finally, the chosen NQRs were to represent the six QRCs situated in different regions in Sweden. The categorisation into different registry types is somewhat arbitrary since some registries, for example the Swedish Hernia Register, the Registry of Gynecological Surgery, and the National Prostate Cancer Registry of Sweden, could be considered both as intervention registries and diagnosis registries (Table 1). The same applies for Senior alert and the Swedish Palliative Care Registry, which could be considered palliative/preventive registries but also intervention registries with a life-long follow-up.

**Table 1:** Overview of the characteristics of the nine National Quality Registries studied

	Intervention	Diagnosis	Palliative/preventive
Acute disease/short care episode	- Swedish National Forensic Psychiatric Registry - Swedish National Registry of Gynecological Surgery - Swedish Hernia Registry		
Chronic disease/life-long follow-up		- Infectious Disease Registry HIV^a^ - Swedish Registry of Congenital Heart Disease - Swedish Follow-up Programme for Cerebral Palsy - National Prostate Cancer Registry of Sweden^a^	- Senior alert - Swedish Palliative^a^Care Registry

^a^Registries at certification level 1 at time of inclusion

### Interview process

Data collection consisted of 18 individual interviews with two persons representing each NQR. Prior to the interviews one researcher (EG) approached registry holders to inform about the study, obtain informed consent to participate and book a time for a telephone interview. The registry holders were also asked to provide a second name from the registry management team who was approached in the same way. All persons approached accepted to be interviewed. Interviews were conducted in Swedish from March to June 2015 by three researchers (MAS, EG or VS). A semi-structured interview guide was developed with open-ended questions concerning how the registry had been working in order to meet the three main areas for the use of NQRs. The questions were formulated in line with the aim and focused on the following themes: action strategies, targets, activities, interaction with others, and facilitating and hindering factors. Interviews lasted from 45 min to 1 h, were recorded and transcribed verbatim.

Documents and websites were used to familiarise ourselves with the NQRs, i.e., by providing descriptions of missions, goals and activities and the scope of the NQR. The NQR organisation and financial reporting was analysed to objectify statements about staffing and financial resources.

The transcribed interviews were analysed iteratively using both conventional and directed content analysis [27]. Two researchers (MAS and VS) separately read all transcripts to obtain an overview and understanding of the data. This was followed by a second reading where the same two researchers, separately, linked the findings to the study aim through coding and subcategorising. The analysis identified activities that the NQR management team focused on in order to reach the goals of the national initiative, i.e., to enhance the use of NQRs in QI, research, and interaction with patients. The information was compiled in a detailed matrix of their vision, activities, actors, and support from QRCs. Validation of the categorisation was done by a third researcher (EG) by going through four interviews (two from MAS and VS, respectively) independently coding them as above. The three researchers then discussed codes in order to ascertain consensus.

As for addressing change barriers and facilitators in healthcare, a directed content analysis approach was applied using Grol and Wensing’s model [28] for the main categories *Innovation, Individual professional, Patient*, *Social context, Organisational context*, and *Economic and political context*. A bottom-up approach was used for identifying and naming subcategories. Illustrative quotes were selected to exemplify each category. The translation of the quotes were made when finalising the manuscript and verified by all authors.

## Results

### Description of the NQRs and their context

The NQRs in this study generally had a governance structure that included a steering committee and a registry operations office. The size of the steering committees varied from 8 to 29 people and were mostly comprised of clinicians, administrators, and patient representatives. Registry office full time staff accounted for between 17 and 83% of the budget. The two registries with the highest percentage (74% and 83%) offered consultation services for their users. The NQRs studied are described in more detail below (Table 2).

**Table 2:** Description of the NQRs included in the study

Registry	Start	Purpose	Target population	Coverage
Infectious Disease Registry HIV	2003	To create good, equitable care regardless of method of infection, gender and care provider by identifying problems and improvement potential.	All HIV-infected patients receiving care in Sweden (more than 6200 patients registered).	Used by all HIV clinics and thereby covering more than 99% of the targeted population.
National Prostate Cancer Registry of Sweden	1996	To monitor time trends and geographical differences with regard to investigation, diagnosis, tumour characteristics and treatment.	All cases of prostate cancer. More than 10,000 patients are registered.	Estimated coverage is 98% of the target population.
Senior alert	2008	To support the preventative care process to prevent falls, pressure ulcers, malnutrition and oral health among the elderly.	All persons 65 years of age or older with any form of contact with health and social care.	Estimated coverage is 55–60% of the target population.
Swedish Follow-up Programme for Cerebral Palsy	2005	To monitor the results of continuous treatment interventions from infancy to adulthood.	People with cerebral palsy in Sweden.	Estimated coverage is 95% of the target population.
Swedish Hernia Registry	1992	To survey the development of hernia surgery in Sweden in terms of methods of repair, waiting times, and results in terms of re-operation or infection.	All adults from 15 years or older with diagnosed inguinal or femoral hernia.	Estimated coverage is 98% of the target population.
Swedish National Forensic Psychiatric Registry	2008	To provide data for improvements and clinical research in order to give the patients safe and reliable care.	All patients handed over by the courts to forensic psychiatric care.	Estimated coverage is 97% of the target population.
Swedish National Registry of Gynecological Surgery	1997	To provide clinics with data for quality assurance, to monitor improvement measures over time, and to conduct research on collected data. The registry also aims to assist the clinics’ documentation procedures and the communication between treating physicians and patients.	All major gynecological procedures.	Coverage is 74–92% depending on type of surgery.
Swedish Palliative Care Registry	2005	To continuously improve end of life care independent of the diagnosis and institution providing the care.	When there is no cure, treatment turns into palliative care which includes support to the family.	More than 60% of all deaths are recorded in the registry.
Swedish Registry of Congenital Heart Disease	1998	To follow patients with congenital heart disease from childhood through adulthood, to obtain as complete information as possible about their natural life course and treatment outcomes.	Around 1000 children yearly are born with a congenital heart disease. More than 40,000 adults in Sweden live with this disease.	Coverage 90%

### Perceived barriers and facilitators for the use of NQRs

As can be seen in Table 3, barriers and facilitators were found in all six areas of Grol and Wensing’s model [28].

**Table 3:** Summary of barriers and facilitators at different levels of health care for using NQRs with illustrative quotes

Levels	Barriers	Facilitators
Innovation	Double administration due to technical constraints or safety and legal barriers in integrating databases. Low data quality due to incorrect data and low coverage. *“You can visit each clinic, connect to the registry and show “this is how you are doing”. We assume of course that the heads of the units do the same.” (Registry team member, NQR5)*	Technical development and increased competence, links to national guidelines, and randomisation possibilities. *“It would be best if a patient with [diagnosis] uses the registry as a place to register and that you thereafter can export these data to the medical records, instead of the way many are testing now, to transfer data from the medical records to the registry.” (Registry holder, NQR5)*
Individual profession	Lack of mandate on behalf of the registry holders to force units to register data or use data for QI work. Lack of interest in registry work on behalf of the healthcare professionals. *“We believe that we make data available, but someone has to request the data.” (Registry holder, NQR7)*	Increased interest in and demand for QI work and research. Linkage between national guidelines and NQRs. Increasing number of enthusiasts with a strong belief in the value of NQRs. *“In order to stimulate as many as possible to get started, we show what has been done, ongoing projects and which areas that have not been researched.” (Registry holder, NQR8)*
Patient	Lack of computer skills and computer access was as well as survey saturation among patients. Not all diseases well suited for self-care. *“… it is not very easy to understand this [output data]; it quite often requires some explanatory text for it to be meaningful and the results understandable.” (Registry holder, NQR5*)	Active patients and patient organisations. Increased use of PROMs and PREMs in QI work and in the patient-caregiver meeting. *“Of course active patients facilitate, an active patient organisation that demands [information]. When the demand increases, the demand on delivery also increases.” (Registry team member, NQR1)*
Social and organisational context^a^	Lack of time, money, and personnel. Problems with incompatible IT-systems and lack of demand from management and principal administrating employers. *“This takes too much administrative time, it takes time away from patient time. This is the most important issue to solve.” (Registry holder, NQR8)*	Recent trends focusing on QI, value-based health care and patient-centred care. QRCs giving support to the production of annual reports, performance of statistical analyses, and QI work. Regular meetings on national or regional levels. *“There has been a paradigm shift towards value-based health care, which fits perfectly with the introduction of PROMs and PREMs… information we can use for important research.” (Registry team member, NQR5)*
Economic and political context	Lack of money and integration between medical records and IT-systems. Top-down steering (such as the national initiative) whereby the enthusiasm for involvement in NQR work is dampened. *“I think that there has sometimes been too much of a top-down perspective on what is to be accomplished and very little of asking questions like: what is it you want to accomplish in these areas? With what do you need help?” (Registry holder, NQR2)*	Contemporary national healthcare investments promoting an increased interest for QI and public benchmarking. Large investments put into the registries. *“In pure economic terms it has of course been very good to have access to a little more money thanks to this initiative, as it has given us the opportunity to develop other parts of the registry.” (Registry team member, NQR3)*

^*a*^ *The social and organisational context overlapped and were therefore placed in one category that included collaboration with other NQRs, QRCs, National Board of Health and Welfare, Swedish Association of Local Authorities and Regions (SALAR) and other national agencies as well as hospitals*

#### Innovation

NQRs were perceived as an innovation with lots of potential provided that data quality is high and the entire target group is covered. NQRs were described to facilitate mapping of new patient groups, (e.g. patients with congenital heart disease who previously did not survive into adulthood) or for long-term follow-up of patients with chronic disorders. The technical development of the registries, e.g. online reporting and the possibility for providers, patients and the general public to access data from registries, were perceived as facilitating improvement and stimulating patient involvement. Moreover, informants described how the use of online reports could enhance meetings with head of units, as well as meetings with patients.

A major barrier mentioned by all informants was “double registration”, where healthcare professionals register (similar or identical) data in electronic health records and in several registries, as systems are not integrated due to technical and/or legal issues. Informants further described a close relation between national evidence-based guidelines and what is entered into the registry, e.g. quality indicators for follow-up and benchmarking of results.

#### Individual professional

Informants expressed the importance of having the health and social care professionals “on board” and motivated when it came to registration and use of data. They had experienced a change in attitude over time and an increasing awareness and interest from the units, which was considered as enabling factors. They also mentioned the importance of having unit managers that value continuous QI and national guidelines. However, as registry holders, they can only provide data from the registries, but do not have the mandate to make the units register data or use data in, for example, their clinical practice.

All registries had an organised process for when researchers and others wanted to extract data. In most cases the steering committee was involved in the decision process, and in many cases they were also involved in the research. Moreover, informants called for the possibility to create a mutual research platform by linking the registry platform with other population-based databases about healthcare consumption, diagnostics, etc.

#### Patients

Informants mentioned the stimulating effect of active patients and patient organisations, and an increased sensitivity on behalf of the registries as to what kind of information patients may need.

One barrier from the patients’ perspective was to understand the registries’ output data, especially for patients with reduced cognitive skills. Making registry data more accessible for lay people was regarded as necessary. Lack of computer skills, survey saturation and the complicated process related to assuring confidentiality were also mentioned.

#### Social and organisational context

The registry holders arranged regular meetings either on a national or regional level where clinicians could meet to discuss results, QI efforts, and research projects. Visits to individual units were also seen as a facilitator in promoting QI efforts and research activities. Other facilitating means mentioned was access to statisticians, enthusiasts and solid routines on the web for data extraction for research. Additionally, recent trends and a parallel focus on for example value-based health care and co-creation of care, were mentioned as drivers for developing patient-reported outcome and experience measures (PROMs and PREMs) and research projects with this focus.

A major barrier was the competition and conflicts regarding how to allocate time, both when it came to time for clinical work and for QI activities. Time allocation between the different NQRs, as some clinics had to register in several NQRs, and the problem with incompatible IT systems were also described as barriers. Some registry holders expressed the need for employers and responsible authorities (county councils, municipalities) to actually request that the units spend time on registering in and using data from NQRs. Such an instruction was perceived to greatly benefit the use of NQRs in all areas.

#### Economic and political context

Financial arrangements, regulations, guidelines, IT-systems, and policies, was seen as both enabling and hindering the use of the NQRs. Other contemporary national healthcare investments have helped to change attitudes and increased interest for QI projects. The large national investments recently put into the registries have mainly been used for allocating time or improving IT-platforms. When asked about the national initiative, informants expressed that the increased funding had made it possible to develop areas that had previously been thought of but not prioritised due to lack of financial resources. However, it was also mentioned that the initiative had used a top-down perspective when setting the goal and follow-up indicators, instead of asking the NQRs what they wanted to focus on within the frame of the initiative’s three focus areas.

### Main activities and interactions with stakeholders in order to enhance the use of NQRs

Three subcategories emerged from the interviews to enhance the use of NQRs for QI, research and interaction with stakeholders: a) activities to ensure that correct and complete data is registered, b) activities to ensure accessible, updated and understandable information available for all stakeholders, and c) activities to promote cooperation with target groups (Table 4). Stakeholders are clinicians, researchers, patients and patient representatives including stakeholders at governmental level.

**Table 4:** Summary of the NQR management’s action strategies to enhance the use of registry data with illustrative quotes

Intention/goal	Activities	Quotes
To ensure that correct and complete data is registered	- Validation of data entered into the registry - Direct data entry - Online access to registered data	*“We get more and more information so that we can evaluate different treatment methods and show which methods are ineffective and try to remove them.” (Registry holder, NQR4)*
To ensure that accessible, updated, and understandable information is available	- Presentation of registry based research at scientific meetings - Presentation of registry reports at regional and/or local meetings with involved professionals - Visits to clinical units to discuss results and registry issues - Annual reports	*“We raise the question and talk about it, describe it on the website, we have it in educations and so on in order to stimulate interest. Our steering committee also knows about it and collects the results. We find many interesting things in need of further research.” (Registry team member, NQR6)*
To intensify cooperation with target groups	- Providing updated information focused on important quality measures that are easy to use in improvement work - Invitations to clinical units to participate in QI collaboratives - Targeted approaches to clinical units in need of support - Having patient representatives in the registry steering committee - Using registry data in the patient consultation - Supporting researchers in accessing and using registry data for research purposes	*“We will contact a number of clinics and say - we see that you are quite obvious having problem with these things, would you like help in structuring an improvement project?” (Registry holder, NQR2)* *“Before a clinical visit, patients are actually sitting at home or at the clinic and using a tablet or a computer to fill in a short survey regarding PREMs,* i.e. *participation and information, and PROMs,* i.e. *potential side effects from treatments. And then he/she comes to me and we click and there it is and then we are interconnected.” (Registry team member, NQR5)*

#### Activities to ensure that correct and complete data is registered

The NQRs’ management team routinely performed time consuming quality controls by comparing patient records with data entered into the registries. However, the informants expressed that the ideal way would be to electronically document directly into the registry. Only one of the nine registries was constructed in this way and the informants from the other registries described various unsuccessful actions to achieve such a solution.

Another activity expressed was online access to registered data. All registries had been involved in such development for several years and were now able to provide online access. One NQR had also developed a much appreciated risk evaluation tool for more valid comparisons between different providers. Verifying and evaluating the evidence base of the data in the registry was said to be in the hands of the researchers assigned to develop new or revise already existing national guidelines. This characterised the work of NQRs, which had either started to check adherence to established guidelines or to construct a knowledge base for development of clinical practice guidelines.

#### Activities to ensure that accessible, updated and understandable information is available for all stakeholders

Informants described different action strategies for communicating the information generated by NQRs to stakeholders. Registry based research results are presented at national and international scientific meetings, and registry reports at national, regional and/or local meetings with involved professions. The regional/local meetings are designed either for managers responsible for the care produced by the units, or for specialists engaged in the care of patients with registry specific diagnoses. The registry-focused meetings concentrate on learning from results and improvements over the past year, and identifying aspects in need of improvement or more research.

All informants underlined the importance of openness when presenting registry based information. Data were presented on unit levels with two exceptions: units with a very low number of patients, and units where the integrity of the patients being treated was at risk. No data referring to individual physicians were openly presented. Informants described plans and activities to improve accessibility of registry based information, for example several language options or pedagogical illustrations. Some described arranging information meetings with and for patients.

Another important service was to provide online, updated and processed reports for clinical users. There is a possibility for the units to follow their quality performance, at any given moment, in relation to set goals. Aggregated data is also provided in the annual reports where the units can compare their results nationally and, in some cases, internationally.

#### Activities to intensify cooperation with stakeholders

Cooperation with healthcare providers, including managers and policymakers, was described in terms of providing updated results of the most important quality measures. Based on key measures, one registry provided support for clinical improvement work through regional representatives who regularly visited all units participating in the registry in the particular region. Another registry sent questionnaires to the participating units asking how the results were used, revealing that some did not make use of the registry data reported to them.

The most common way to offer collaboration was to invite healthcare units to participate in an improvement project set up by the registry management, often with support from a QRC. A few informants described more targeted strategies where units in need of improving their performance were identified and offered support in setting up and pursuing improvement projects. Some registries offered consultation services directed at physicians in need of discussing a clinical problem with a colleague.

Informants described various strategies for cooperation with patients. For registries with existing patient associations a common feature was to have one or two representatives in the steering committee from that association. When no relevant patient associations were established there were alternative ways to ensure patient representation. Registry representatives were also regularly invited to patient associations’ meetings to present current registry information. Patient cooperation and participation in the design of questionnaires, information materials and guidelines was described as an important feature in the use of NQRs for clinical development.

The use of NQR data in patient consultations was described as a very important measure to support patient engagement (e.g. support self-care) and thus promote patient cooperation in the development of registries. Informants described how patient-specific data, including questionnaires filled in by the patient, were used in patient meetings to discuss treatment results and disease control.

All informants underlined the importance of cooperating with researchers and using registry data for scientific purposes. Research questions were often raised within the registry itself and handled by one or two researchers in the steering committee or were advertised and presented at various user meetings as suggestion for scientific studies. In recent years, the informants confirmed an explosive interest concerning data access for research purposes. This was said to be due to the stimulating effects on both established and potential researchers by well-developed routines for accessing registry data.

### Variation between the different types of NQRs in regard of their use

When informants were asked if the type of registry (intervention, diagnosis or palliative/preventive) or type of care process (acute/short or chronic/life-long) impacted their ability to reach the goals of the national initiative many reported they had not reflected on this.

Long-term and chronic condition registries were said to create greater interest among patients (and care providers). Depending on what kind of care the registry covers, different prerequisites were described in terms of communication and adjustment to target groups. For example, a registry that only covered a few specialist clinics versus registries with many contacts in hospitals, social care and home care would require different strategies.

Patient interaction was more likely to be captured by long-term care registries since they generally communicate with patient organisations and active patients living with the condition. Some informants thought that patient interaction might be more difficult in registries covering for example elderly and/or dementia care, intensive care (sometimes unconscious patients), psychiatry or defined interventions (diagnostic or therapeutic). Informants pointed out that accessible online data was more difficult to provide for registries where results are not immediate or intuitive, or when it takes time for results to appear.

## Discussion

This study, based on information from nine well-developed Swedish NQRs, has shown that they are perceived as an innovation with high potential for QI, research and knowledge development, and for patient involvement, but that the use for these purposes is still in its infancy.

One major facilitator for enhancing the use of the NQRs was having sufficient funding. The recent national investment initiative enabled the development of areas previously identified by the NQR management teams. The relatively recent focus on value-based health care was pointed out as an especially positive facilitator, as well as other contemporary national healthcare investments aiming at QI and public benchmarking. The current focus on value-based health care has also stimulated patient involvement and the increased use of PROMs and PREMs in registries. However, real patient involvement will require more direct interaction with both individual patients and groups of patients. For example, patient and patient representatives can add important perspectives of the care process, choice of variables, and data presentation if involved in the NQR steering group or in focus groups. This is something that has been encouraged by the national initiative, but a lot remains to be done. During patient consultation, clinical measures are complemented with patient dependent measures providing opportunities for a shared and more holistic overview of the patient’s health status, which can facilitate patient interaction and co-production of care [3, 29]. It will, however, depend on whether patients are invited to participate and able to engage.

A major barrier was users having to perform double registration due to shortcomings in design of the digital systems which has also been pointed out in other studies [11, 13]. Less capacity is then left for actually using the data for e.g. QI work, and the situation makes patient involvement and research more difficult [10, 19]. Another barrier was the lack of authority on behalf of the registry management teams to request participation in the registry as well as in QI activities based on registry outcomes. The registry management teams were aware of the fact that they do not have the mandate to “force” the units to use the NQRs. Their option to impact healthcare professionals is rather to use “peer pressure, shaming and a sense of moral responsibility” described as potential compliance mechanisms [30]. Studies have revealed a shortage of supportive structure in terms of committed and accountable leadership at the political and clinical levels [13, 30] as well as a lack of engagement on behalf of the local staff and managers in using quality registries for QI work [4, 19, 20]. The lack of formal mandate was also pointed out by QRCs as a main challenge when supporting NQRs to be used in healthcare organisations [25].

In fact, one could say that this whole initiative lacks a multilevel approach in accordance with Ferlie & Shortell’s Framework for change, which is characterised by four essential core properties needed in order to really achieve change and thereby improved quality of care. These core properties are: “1) leadership at all levels; 2) a pervasive culture that supports learning throughout the care process; 3) an emphasis on the development of effective teams; and 4) greater use of information technologies for both continuous improvement work and external accountability” [31]. The national initiative has so far mostly been focused on number four, through increasing the use of NQRs whereas less emphasis has been on numbers one to three, which concern leadership, change of culture and behaviours. Furthermore, it is important to understand that barriers to change exist at different levels; both individual, team, organisation, and system levels. Strategies for change need to be tailored for each and every one of these levels [31, 32].

Indeed, the great variety of strategies among quality registries to achieve the three goals of the national initiative, i.e., to increase their use for QI, research, and patient engagement, may reflect that more emphasis was put, on the national level, on policy formulation than policy implementation. We find the model for evidence-informed policy formulation and implementation, presented by Strehlenert et al. [33] enlightening in this respect. The national initiative did have an element of “capacity building”, as six regional QRCs were established to support NQRs. In terms of implementation, the annual cycle of funding application and reporting acted as “raising awareness” among NQRs about the targets. But there was less emphasis on supporting the “adoption” of strategies among NQRs, as well as “implementation” and “maintenance” in terms of integrating the NQR strategies into their ordinary operations. More focus on those elements of policy implementation could have improved the efforts of NQRs to reach the goals, and, subsequently, increased the success of the national initiative.

The registry management teams used three different strategies to enhance the use of NQRs within the three focus areas: ensuring registering of correct and complete data, ensuring updated and understandable information available for all stakeholders, and intensifying cooperation with relevant stakeholders. However, the need of improving methodological competence among registry researchers, especially concerning use of epidemiologic methods, did not surface as a chosen strategy to enhance the use of NQRs. This has been emphasized by Adami and Hernan [4] as a crucial strategy for increasing the value of NQRs within research. Additionally, the possibilities of using NQRs for randomised trials – even mega trials as has been pointed out by Lauer et al. [34] – was not mentioned as a possible strategy. There is a risk that the NQRs will remain an “untapped resource” when it comes to using NQRs for research purposes as long as such strategic approaches are overlooked [4].

Furthermore, there were no examples in our study, that the registry management teams offered support for benchmarking processes. Such processes differ from standard quality improvement processes by “the dynamic of comparing and learning from each other” [35] and the “comparative evaluation and identification of the underlying causes leading to high levels of performance” [35, 36]. The strategies mentioned to support the improvement of the results of less successful organisations were described in terms of classical internal quality improvement projects without the elements of benchmarking, i.e., learning from those with the best results in order to understand what makes their organisations so successful. Such initiatives would not only clarify the need for change of practice but also contribute to the insight into the contextual barriers (organisational, social, and professional) in their own organisation that counteract desirable changes [32].

The characteristics of the NQRs have an influence on their use. This study showed that the type of registry and its potential use may be connected to what kind of data is registered, the specific condition or type of care process followed, whether it is acute/short or chronic/life-long intervention, and if the registry focuses on diagnosis or on risk assessments and prevention. This has also been observed by Fredriksson et al. [14] studying the usefulness of using data from the NQRs for QI. These observations should guide further studies on strategies to promote the use of NQRs.

### Methodological considerations

Most of the previous studies involving NQRs have focused on one registry or addressed the NQRs as a phenomenon (e.g. [8, 11, 13]). This study is based on nine NQRs out of approximately 100. We used sampling methods aiming at maximising variation and registry purpose. However, generalisation to all registries should be made with caution. The registries are managed by a limited number of people and out of them we chose the registry holder who in turn chose one of his or her colleagues that had knowledge on the use of the registry in the three focus areas. Interviews with e.g. non-registry staff could possibly have provided more variation in views. To ensure reliability in analyses three researchers were involved and we checked for validity, in both a-priori categories and inductive analyses. Websites and documents complemented the interview, but they risk being biased by efforts to present NQRs in a favourable way.

## Conclusions

This study has shown that the potential use of NQRs for QI, research and interaction with stakeholders is related to the funding of the registries and that a recent national investment initiative contributed to already ongoing purposeful work to strengthen what was seen as facilitating factors (such as technical development) and likewise counteract perceived barriers (such as lack of authority). Whether this in fact led to the intended goal of the initiative: increased use of NQRs for QI, research and interaction with stakeholders, has not been examined in this study.

The way the registries in fact are used for these purposes is a question for the end-users, i.e. the clinicians, the researchers and the patients. For future national investment initiatives, where an increased use of the registries is desirable, the needs of these end-users must be in focus. Since there is a great variation between different types of registries it is reasonable to believe that there is variation in prerequisites when it comes to enhancing their use for QI, research and interaction with stakeholders. However, the end-users of the different registries may all have in common a need for information on and training in the methodology of registry based research as well as benchmarking methodology, i.e. how to learn from best practices, and how to involve patients and families in patient centred care.

## Abbreviations

NQR: National Quality Registry
PREM: Patient-reported Experience Measure
PROM: Patient-reported Outcome Measure
QI: Quality Improvement
QRC: Quality Registry Centres
SALAR: Swedish Association for Local Authorities and Regions
